# Supplementary material for: Phase I Targeted Combination Trial of Sorafenib and GW5074 in Patients with Advanced Refractory Solid Tumors
Source: J Clin Med. 2022 Apr 14;11(8):2183. doi: 10.3390/jcm11082183 (PMC9031611; doi:10.3390/jcm11082183)
Supplement: Supplementary file 1 [file jcm-11-02183-s001.zip › jcm-1645752-supplementary.pdf]

Table S1. The criteria of DLT.

* Any death not clearly due to the underlying disease or extraneous causes
* Non-hematologic toxicity:<br>● Grade 3 or higher<br>● Hy’s law
* Hematologic toxicity:<br>● Grade 4 neutropenia or thrombocytopenia >7 days<br>● Grade 3 thrombocytopenia with bleeding<br>● Grade ≥3 anemia<br>● Neutropenic fever
* The DLT definition excludes:<br>● Grade 3 nausea/vomiting or diarrhea <72 hours with adequate antiemetic and other supportive care<br>● Grade 3 fatigue <1 week<br>● ≥Grade 3 electrolyte abnormality that lasts <24 to 72 hours, is not clinically complicated, and resolves spontaneously or responds to conventional medical interventions<br>● ≥Grade 3 amylase or lipase that is not associated with symptoms or clinical manifestations of pancreatitis

The DLT criteria are defined as the following events that are possibly, probably, or definitely related to the investigation drug.
